# Angiotensin-(1-7)/Angiotensin-Converting Enzyme 2/Mas Receptor Axis and Related Mechanisms

**DOI:** 10.1155/2012/690785

**Published:** 2012-04-09

**Authors:** Anderson J. Ferreira, Robson A. S. Santos, Mohan K. Raizada

**Affiliations:** ^1^Department of Morphology, Federal University of Minas Gerais, 31270-901 Belo Horizonte, MG, Brazil; ^2^Department of Physiology and Biophysics, Federal University of Minas Gerais, 31270-901 Belo Horizonte, MG, Brazil; ^3^Department of Physiology and Functional Genomics, College of Medicine, University of Florida, P.O. Box 100274, Gainesville, FL 32610, USA

Cardiovascular disease is America's leading health problem and the leading cause of death. One person in three suffers from some form of cardiovascular disease. There is an urgent need to develop new therapeutic strategies to improve the cardiovascular diseases outcome [1]. Very recent studies have demonstrated that the heptapeptide angiotensin (Ang)-(1-7) holds important cardiovascular beneficial effects [2]. These actions are generally mediated by activation of the G protein-coupled receptor Mas [3]. As a result of these novel findings, Ang-(1-7), in combination with Mas and angiotensin-converting enzyme (ACE) 2 [4, 5], the main enzyme involved in its formation, is thought to compose a cardioprotective branch within the renin-angiotensin system (RAS), which balances the ACE/Ang II/AT_1_ receptor axis effects [6]. Consequently, new therapeutic approaches targeting the ACE2/Ang-(1-7)/Mas axis have been proposed.

In view of these new and exciting findings, we proposed a special issue in the International Journal of Hypertension. Our main objective was to compile original research articles as well as review articles from outstanding investigators around the World contributing to the continued efforts to understand the role of this axis in the cardiovascular system. Particularly, our intention was to give some insights into the advances in therapeutic approaches based on the ACE2/Ang-(1-7)/Mas axis to treat cardiovascular diseases. 

This special issue is composed of 15 papers (original articles and reviews). Importantly, part of them was presented in the VIII Vasoactive Peptides Symposium which was held in Brazil in April, 2011. This meeting was a unique opportunity for clinicians and researchers to exchange their experience within the cardiovascular field focusing on the RAS. 

The paper provided by N. E. Clarke and J. Turner is an overview of the biological roles of ACE2. They focused on the entrance of recombinant human ACE2 (rhACE2) into clinical trials discussing the potential use of this enzyme as a therapeutic strategy. A. J. Ferreira et al. expand this view to the whole ACE2/Ang-(1-7)/Mas axis highlighting the initiatives to develop potential therapeutic approaches based on this axis. Other three papers are original articles evaluating examples of these strategies. M. Durik et al. investigated the effects of a stabilized, thioether-bridged analogue of Ang-(1-7) called cyclic Ang-(1-7) [cAng-(1-7)] in rat model of myocardial infarction (MI). They found that MI increased the heart weight and myocyte size which was restored by cAng-(1-7) to sham levels. In addition, cAng-(1-7) lowered left ventricular end-diastolic pressure and improved endothelial function. The second strategy discussed in this special issue was the Ang-(1-7) included in hydroxypropyl *β*-cyclodextrin (HP*β*CD). F. D. Marques et al. evaluated the chronic cardiac effects of this compound in infarcted rats. Once-a-day oral HP*β*CD/Ang-(1-7) administration improved the cardiac function and reduced the deleterious effects induced by MI on TGF-*β* and collagen type I expression. Finally, the antiproliferative effects of the nonpeptide AVE 0991, an agonist of Mas, were investigated by C. Sheng-Long et al. It was observed that AVE 0991 attenuates the Ang II-induced vascular smooth muscle cells proliferation in a dose-dependent fashion and that this effect is associated with the Mas/heme oxygenase-1/p38 MAPK signaling pathway.

In this special issue, the role of the ACE2/Ang-(1-7)/Mas axis in the cardiovascular function was extensively discussed in review articles and new findings regarding this axis were presented. E. R. M. Gomes et al. revised the actual knowledge about Ang-(1-7)-mediated signaling in cardiac cells, as well as the discoveries made in cardiomyocyte physiology through the use of genetic approaches. Interestingly, M. Poglitsch et al. reported significant differences in the conversion rates of recombinant human and recombinant murine ACE2 (rhACE2 and rmACE2, resp.) for diverse natural peptide substrates in plasma samples. They found species-specific differences in substrate specificities, probably leading to functional differences in the alternative axis of the RAS. Specially, in contrast to rhACE2, rmACE2 is substantially less potent in transforming Ang-(1-10) to Ang-(1-9). Of note, this enzymatic pathway was reviewed by M. P. Ocaranza and J. E. Jalil. They presented current experimental evidence suggesting that activation of the ACE2/Ang-(1-9) pathway might protect the heart and vessels from adverse cardiovascular remodeling in hypertension and in heart failure. 

In addition to the cardiovascular system, some insights were given in the renal system in this special issue. S. V. B. Pinheiro and A. C. Simões e Silva highlighted the current understanding of the ACE2/Ang-(1-7)/Mas axis in renal physiology and in the pathogenesis of primary hypertension and chronic kidney disease. Also, L. C. Barroso et al. presented some data demonstrating the renal effects of acute administration of AVE 0991 in a murine model of renal ischemia/reperfusion. Administration of AVE 0991 promoted renoprotective effects evidenced by improvement of renal function, decreased tissue injury, prevention of local and remote leukocyte infiltration, and release of the chemokine CXCL1. 

In the past few years, the understanding of the RAS has improved significantly. This is not only a consequence of the identification of novel members, but also due to advances in the knowledge concerning the role of these components in many tissues and cells [2, 6]. Here, M. A. F. Godoy et al. provided an overview of the functional role and the molecular pathways involved in the biosynthesis and activity of Ang-(1-7) in diverse systems, including its actions in gastrointestinal smooth muscles. Also, S. E. Thatcher et al. evaluated the effects of ACE2 deficiency in bone marrow-derived stem cells on adipose inflammation and glucose tolerance in mice fed a high-fat diet. They concluded that ACE2 deficiency in these cells promotes inflammation in adipose tissue and augments obesity-induced glucose intolerance. Interestingly, J. W. Prokop et al. demonstrated that many different genes participating in the RAS can be affected by testis determining protein (SRY), apparently in coordinated fashion, to produce more Ang II and less Ang-(1-7). 

In certain circumstances and in some tissues, AT_2_ receptors appear to be involved in the Ang-(1-7) effects [7]. Furthermore, physical interaction between Mas and AT_2_ in selected tissues such as heart has been suggested as a putative mechanism for Ang-(1-7) actions [8]. In this special issue, R. E. Widdop's group presents an original article demonstrating the relationship between Ang-(1-7) and AT_2_ receptors. S. Bosnyak et al. reported that the hypotensive effect of Ang-(1-7) was dependent on the background dose of candesartan and that this effect was reversed by AT_2_ receptor blockade. In aged rats, the depressor effect of Ang-(1-7) was evident but was inhibited by either AT_2_ or Mas receptors blockade. Furthermore, E. S. Jones et al. reported that AT_2_ receptor stimulation does not significantly influence the antihypertensive effect of chronic AT_1_ receptor blockade but plays a role in the regulation of vascular structure, as well as in cardiac perivascular fibrosis. 

Thus, we hope that this special issue is able to make a picture of the role of the ACE2/Ang-(1-7)/Mas axis and of the potential therapeutic of this concept for treating cardiovascular and related diseases. Our expectation is that this special issue might serve as an important reference to scientists and physicians to update their knowledge about this contemporary theme. 

*Anderson J. Ferreira* *Anderson J. Ferreira* *Robson A. S. Santos* *Robson A. S. Santos* *Mohan K. Raizada* *Mohan K. Raizada*
